# Machine learning-derived identification of an obesity and lipid metabolism-related genes signature for the diagnosis and molecular typing of acute myocardial infarction

**DOI:** 10.3389/fcvm.2026.1694872

**Published:** 2026-03-27

**Authors:** Jianhua Li, Zixin Xue, Min Zhang, Feng Lu, Shaoping Ji

**Affiliations:** 1 Center for Molecular Medicine, Zhengzhou Health College, Zhengzhou, Henan, China; 2 Department of Immunology, School of Basic Medical Sciences, Henan University, Kaifeng, China; 3 Department of Biochemistry and Molecular Biology, School of Basic Medical Sciences, Henan University, Kaifeng, China

**Keywords:** acute myocardial infarction, diagnostic model, immune infiltration, machine learning, obesity and lipid metabolism-related genes

## Abstract

**Background:**

Acute myocardial infarction (AMI) ranks among the leading causes of death globally and is linked to obesity and the metabolism of lipids. The objective of this research was to develop an innovative predictive model utilizing obesity and lipid metabolism-related genes (OLMRGs) to facilitate the diagnosis and molecular typing of AMI.

**Methods:**

Microarray data were obtained from the Gene Expression Omnibus (GEO) repository, while OLMRGs were extracted from the GeneCards and GSEA databases. Important signature genes were pinpointed utilizing univariate regression analysis, LASSO regression, Random Forest, and SVM algorithms. A diagnostic model was then developed using logistic regression. The model's diagnostic efficacy was subsequently confirmed in the validation set GSE59867. Immune infiltration levels were assessed via ssGSEA, and the key genes were validated using RT-qPCR.

**Results:**

An obesity and lipid metabolism-related genes signature, consisting of five genes (IL1RN, SERPINA1, CEBPB, NFKBIA, and VNN1), was developed as a diagnostic biomarker for AMI (AUC = 0.827) and corroborated in the GSE59876 dataset (AUC = 0.870). The diagnostic model revealed comparisons between groups at high and low risk, identifying twenty-four unique immune cell types alongside nineteen distinct immune functions. Additionally, validation with RT-qPCR confirmed the differential expression of these five signature genes in both AMI and control samples.

**Conclusion:**

The novel five-gene signature may act as a new biomarker indicating the presence of AMI, providing fresh perspectives for AMI diagnosis and molecular classification.

## Introduction

Acute myocardial infarction (AMI) ranks among the primary causes of death attributed to cardiovascular diseases globally (1, 2). Although advancements in pharmacological treatments for AMI patients have been made, the overall outlook for these individuals is often not favorable (3). The onset of AMI is primarily precipitated by acute coronary syndrome, chiefly arising from plaque formation or intravascular clots, which results in myocardial hypoxia, tissue death, and extensive myocardial injury. Some AMIs may initially present without symptoms; thus, prompt diagnosis and timely interventional care are essential for improving outcomes and decreasing mortality related to AMI (4). At present, biomarkers are crucial in facilitating the diagnosis of AMI (5, 6). The use of cardiac biomarkers, such as cardiac troponin I and T, has been extensively adopted in the clinical diagnosis of AMI (7, 8). Nevertheless, relying on these biomarkers for AMI diagnosis remains inadequate due to their specificity and sensitivity limitations (9, 10). It is also noteworthy that an effective method for early detection of AMI is still lacking. Consequently, it is vital to discover new biomarkers for diagnosing AMI. Additionally, the intricate etiology and pathophysiological mechanisms associated with AMI contribute to substantial variability in prognosis among patients. The urgency for molecular subtyping of AMI patients is becoming increasingly evident, as it can yield important insights into the underlying pathophysiological processes of the condition and identify specific biomarkers and genetic differences that may influence severity, prognosis, and treatment response. Moreover, molecular subtyping can aid in creating novel therapeutic strategies and pinpointing patients who could benefit from targeted interventions, thus improving the overall management of AMI.

Numerous investigations have been conducted to gain a deeper understanding of the mechanisms underlying the pathogenesis of AMI. Among these findings, it has been highlighted that obesity and lipid metabolism significantly contribute to cardiovascular disease (CVD), including AMI (11). Obesity directly and indirectly facilitates the progression of CVD (12). Increased adiposity leads to endothelial dysfunction, remodeling of small vessels, and cardiomyocyte toxicity, which can promote conditions such as atherosclerotic coronary heart disease, arrhythmias, cardiomyopathy, and congestive heart failure (13). Furthermore, obesity serves as a significant risk factor for the development of various established cardiovascular risk factors, such as dyslipidemia, hypertension, type Ⅱ diabetes, and chronic kidney disease (14, 15). The relationship between lipid metabolism and AMI is particularly strong (16). Under normal physiological circumstances, fatty acid β-oxidation provides approximately 50%–70% of the energy required by the heart (17). Conversely, elevated levels of serum cholesterol and free fatty acids (FFA) are recognized as risk factors for cardiovascular disease and stand as independent predictors of cardiovascular mortality (18). An accumulation of excessive low-density lipoprotein cholesterol (LDL-C) in arteries results in plaque formation, heightening the risk of AMI. It has been observed that increased lipid availability can exacerbate ischemia-related cardiac dysfunction and diminish the efficiency of myocardial mitochondria (19). In mouse models of myocardial infarction, a deficiency in Apolipoprotein E results in the production of excess neutrophil extracellular traps, exacerbating myocardial damage (20). Despite this, so far, research focused specifically on the roles of obesity and lipid metabolism-related genes (OLMRGs) concerning the onset, development, diagnosis, and prognosis of AMI remains limited.

Currently, bioinformatics analysis facilitates a comprehensive understanding of complex physiological processes through the extraction and interpretation of biological data obtained from public datasets. This approach has proven to be a valuable tool, effectively applied to a variety of diseases, such as cardiovascular disorders (21, 22). Conventional approaches generally concentrate on a single gene or a limited number of proteins; however, bioinformatics allows us to analyze intricate biological systems as cohesive entities. For example, utilizing multiple algorithms such as weighted gene co-expression network analysis (WGCNA), logistic regression, random forest, support vector machines (SVM), and Mendelian randomization analysis, researchers can uncover potential biomarkers and establish reliable predictive models for AMI or MI diagnosis (23–26). In addition, employing gene expression profiles enables the classification of DVD patients into distinct molecular subtypes, each exhibiting unique characteristics (27, 28), which might serve as valuable data for making targeted and personalized treatment decisions.

In this study, we aimed to identify an obesity and lipid metabolism-related genes signature (OLMRGS) for the diagnosis of AMI. We sourced gene expression profiles associated with AMI and control samples from the GEO database, and subsequently conducted an analysis of differentially expressed genes (DEGs). Through an extensive bioinformatics approach, we pinpointed key OLMRGs and established a new diagnostic model which was validated to examine the significance of the OLMRGS in AMI diagnosis. Furthermore, we identified two molecular subtypes linked to obesity and lipid metabolism that exhibited distinct immune infiltration patterns. Overall, our innovative findings present a highly effective OLMRGS for the identification and classification of AMI patients, potentially enhancing precision and individualized treatment strategies.

## Materials and methods

### Data collection and preparation

Datasets were retrieved from the publicly available Gene Expression Omnibus database (https://www.ncbi.nlm.nih.gov/geo/) using the search terms “acute myocardial infarction” or “AMI”, “array” type, and “Homo sapiens.” The inclusion criteria for datasets required that each group contain at least five patients and five controls, and that gene symbols and Entrez IDs were available in the annotated platforms (GPL). Ultimately, five datasets were selected (Supplementary Table S1). To create a metadata file, datasets GSE62646, GSE66360, GSE60993, and GSE48060, comprising 115 AMI samples and 92 control samples, were combined. This aggregated metadata served as the training cohort for the integrated bioinformatics analysis. In addition, GSE59867, which contains 111 AMI samples and 46 controls, was utilized as an independent testing cohort. The data preprocessing and removal of batch effects were achieved through the application of the ComBat function from the SVA package (29). Obesity-related genes (ORGs) were obtained from the GeneCards database (https://www.genecards.org/) by searching the term “obesity” with a relevance score criterion of ≥5 (Supplementary Table S2). Additionally, a total of 1,045 lipid metabolism-related genes (LMRGs) were compiled from the Gene Set Enrichment Analysis database (https://www.gsea-msigdb.org/gsea/msigdb/) and previous studies (Supplementary Table S3) (30–32). The analysis of differentially expressed genes (DEGs) between AMI and control groups was carried out using the “limma” R package, employing a significance threshold of a *p*-value <0.05 and | log2 (fold change) | ≥0.585. The differentially expressed obesity and lipid metabolism-related genes (DE-OLMRGs) were identified by intersecting the DEGs with ORGs and LMRGs.

### Analysis of functional enrichment

DEGs were assessed for enrichment in Gene Ontology (GO) and Kyoto Encyclopedia of Genes and Genomes (KEGG) pathways through the R package “clusterProfiler” (33), employing the reference gene set “c2.cp.v7.2.symbols.gmt.” A significance threshold was established at *p* < 0.05, and the outcomes were illustrated utilizing the R package “ggplot2” (34).

### Screening of candidate signature genes and development of diagnostic model associated with DE-OLMRGs

We employed several machine learning techniques, such as logistic regression, SVM (35), least absolute shrinkage and selection operator (LASSO) regression (24), and random forests (RF), to identify potential signature genes for the development of a diagnostic model for AMI. This methodology was supported by various studies of notable scientific significance within the bioinformatics domain (36, 37). By integrating the findings from the four algorithms, we derived a set of common candidate feature genes. These genes were subsequently validated using the GSE59867 dataset and RT-qPCR experiments. From the training and testing datasets, we extracted the expression levels of the feature genes, and calculated the risk score for each sample using the following formula: Risk score = *β*_0_+ $\overset{n}{\underset{i}{\sum }}\;\mathrm{Coefi}$ *x_i_. In this formula, *β*_0_ represents the constant, Coef_i_ indicates the logistic regression coefficient for *i* gene, *x_i_* denotes the expression measurement of the *i* gene, and *n* reflects the total number of genes incorporated in the regression model. To evaluate the diagnostic performance of the feature genes, we generated a receiver operating characteristic (ROC) curve using the pROC package. Ultimately, we created a nomogram to represent the diagnostic model and predict the likelihood of AMI based on the selected signature genes with the assistance of the “rms” package. We also plotted the calibration curve and conducted decision curve analysis to evaluate the predictions and effectiveness of the nomogram model.

### Identification of molecular subtypes and analysis of the immune microenvironment

In the training cohort, AMI samples were categorized into low- and high-risk groups based on the median of the risk scores (28). The fraction of 28 immune cells and the score for 29 immune functions in each sample were computed using single-sample Gene Set Enrichment Analysis (ssGSEA), which relies on gene expression patterns. Differences in immune cell populations and immune functions across various subgroups were assessed and depicted through violin plots or box plots. Furthermore, Spearman's rank correlation analysis was employed in R software to examine the relationship between the identified signature gene biomarkers and the levels of infiltrating immune cells. The resulting associations were visualized using the “ggplot2” package for charting.

### Analysis of pathway enrichment utilizing gene set enrichment analysis (GSEA)

Differentially expressed genes between low-risk and high-risk cohorts were analyzed for KEGG pathway enrichment through the GSEA approach (38). A significance threshold of *p* < 0.05 was utilized for determining significant pathways.

### Revers transcription-quantitative polymerase chain reaction (RT-qPCR)

Blood samples were obtained from seven individuals identified with AMI and from seven healthy control subjects at Huaihe Hospital of Henan University, China. Participants were excluded from the study based on several criteria, including a diagnosis of cancer, severe infectious diseases, advanced liver and kidney failure, disorders of the blood, autoimmune conditions, and any previous history of cardiovascular disease (see Supplementary Table S4). Informed consent was obtained from all subjects, including both the patients and the control group, prior to sample acquisition. The procedures involving human blood were conducted in accordance with the ethical standards outlined by the Declaration of Helsinki and received the necessary approval from the Medical School's Ethics Committee at Henan University, China (HUSOM-2018-282). Total RNA isolation from peripheral blood was achieved by modifying the RNeasy Mini Kit (Qiagen, Cat No:74104) protocol. Quantitative reverse transcription PCR analysis were performed using standard techniques. Relevant details have been provided in earlier publications (Supplementary Materials and Methods) (39, 40). Gene expression quantification was performed utilizing the 2^−ΔΔCt^ methodology, with GAPDH serving as the internal control. Primer sequences are listed in Supplementary Table S5.

### Statistical analysis

Analyses were performed employing R (version 4.4.2) or the SangerBox platform (http://sangerbox.com/), with GraphPad utilized for managing experimental data. A *p* value < 0.05 was considered statistically significant, unless stated otherwise.

## Results

### Screening of differentially expressed obesity and lipid metabolism-related genes (DE-OLMRGs) and functional enrichment analysis

**Figure 1:**
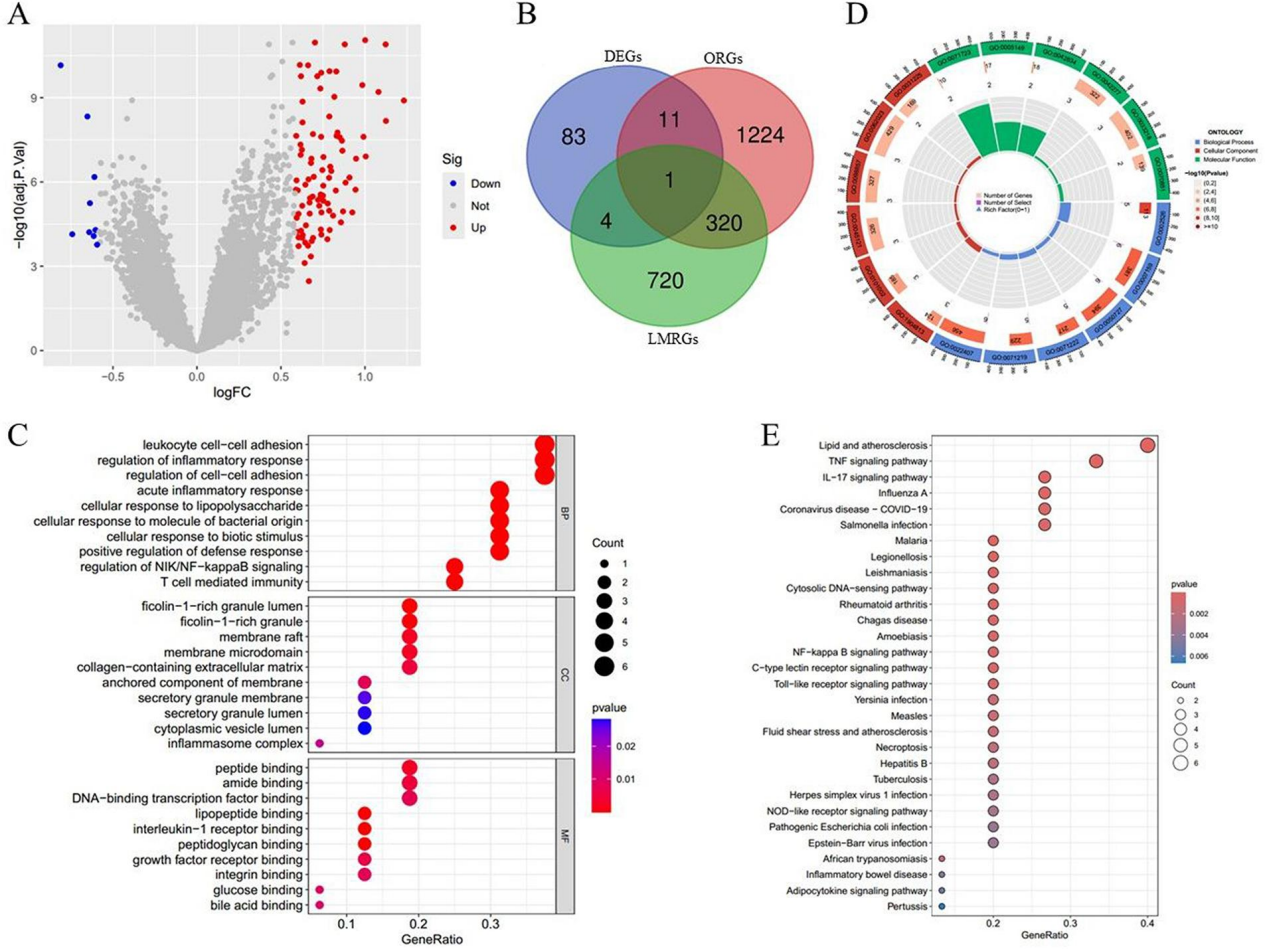
Differentially expressed obesity and lipid metabolism-related genes (DE-OLMRGs) and function enrichment analysis in acute myocardial infarction (AMI) and control samples. **(A)** The volcano map of differentially expressed genes (DEGs) between AMI and control samples. **(B)** Identification of 16 DE-OLMRGs by intersecting the DEGs with LMRGs and ORGs. **(C,D)** Bubble plots **(C)** and circle plots **(D)** of the GO enrichment analysis of the 16 DE-OLMRGs. **(E)** KEGG pathway analysis of the 16 DE-OLMRGs. DEGs, differentially expressed genes; LMRGs, lipid metabolism-related genes; ORGs, obesity-related genes; GO, Gene Ontology; KEGG, Kyoto Encyclopedia of Genes and Genomes.

In this research, we utilized four GEO datasets: GSE48060, GSE60993, GES66360, and GSE62646. The boxplot (Supplementary Figure S1A), density plot (Supplementary Figure S1C), and principal component map (Supplementary Figure S1E) for these datasets demonstrated considerable variation in sample distributions, suggesting the presence of a batch effect. Upon applying the empirical Bayesian method known as COMBAT to mitigate this effect, the data distributions across the datasets began to align more closely (Supplementary Figures S1B,D,F), reflecting an effective removal of the batch effect. Subsequently, we conducted a differential expression analysis using the “limma” package in R software, resulting in the identification of 99 differentially expressed genes (DEGs), comprising 90 genes that were upregulated and 9 that were downregulated (Supplementary Table S6). In the volcano plot (Figure 1A), the downregulated and upregulated genes exhibited a significant distinction. By intersecting these 99 differentially expressed genes with 1,556 genes related to obesity and 1,045 genes pertinent to lipid metabolism, we identified 16 DE-OLMRGs (Figure 1B). To investigate the biological processes and signaling pathways associated with DE-OLMRGs, we executed GO and KEGG analyses. The ten most significant GO terms revealed that these DE-OLMRGs were prominently implicated in biological processes such as leukocyte cell-cell adhesion, regulation of inflammatory response, and acute inflammatory response (Figures 1C,D). The KEGG analysis highlighted that pathways related to lipid metabolism, atherosclerosis, TNF signaling, and IL-17 signaling were connected to DE-OLMRGs (Figure 1E).

### Identification and validation of diagnostic signature genes

**Figure 2:**
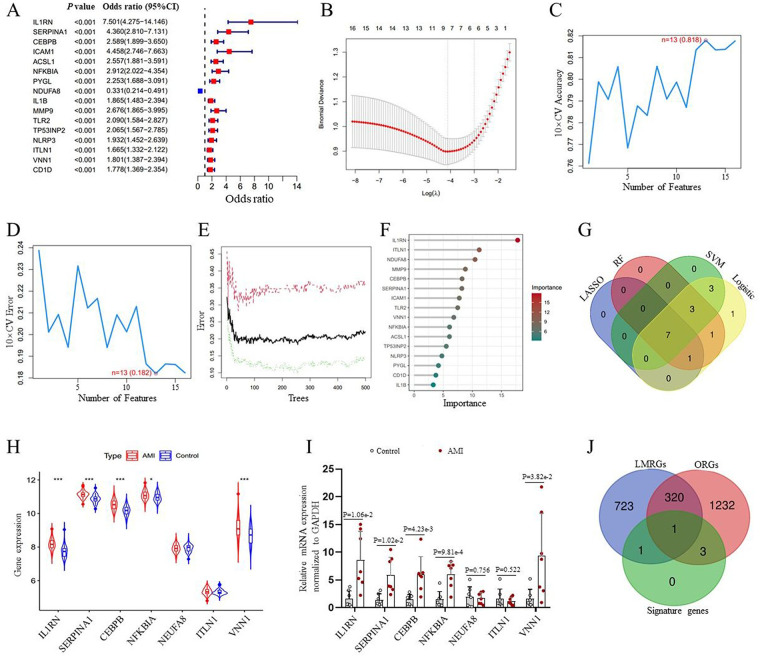
Screening process of diagnostic signature genes for AMI. **(A)** Forest blot of univariate logistic regression analysis. **(B)** The outcomes of the least absolute shrinkage and selection operator (LASSO) analysis. **(C,D)** A plot of biomarkers selection utilizing the support vector machine-recursive feature elimination (SVM-RFE) algorithm. **(E)** Identification of the AMI-specific genes using the random forest (RF) approach. **(F)** The most importance genes selected by the RF with the *x*-axis representing the importance index and the *y*-axis listing the respective genes. **(G)** A Venn diagram illustrating seven candidate diagnostic markers that are shared among the logistic regression, LASSO, SVM-RFE, and RF analyses. **(H)** Validation of the expression levels of candidate diagnostic markers using the GSE59867 dataset. **(I)** Candidate diagnostic markers were validated in peripheral blood samples from AMI patients and healthy controls through RT-qPCR. **(J)** A Venn diagram depicting the distribution of diagnostic signature genes. LMRGs, lipid metabolism-related genes; ORGs, obesity-related genes. *p < 0.05, ***p < 0.001.

To uncover genes with potential diagnostic applications, all 16 DE-OLMRGs underwent univariate logistic regression (Figure 2A). Subsequently, three distinct algorithms were employed to refine the search for potential biomarkers. The LASSO regression algorithm was utilized to narrow down the DE-OLMRGs, leading to the discovery of 8 variables identified as diagnostic biomarkers for AMI (Figure 2B). Through the SVM-RFE algorithm, a subset consisting of 13 features among the DE-OLMRGs was recognized (Figures 2C,D). Random forest analysis further revealed 12 genes considered to have diagnostic significance (Figures 2E,F). The intersection of the results from the four machine learning algorithms culminated in the identification of seven pivotal genes: IL1RN, SERPINA1, CEBPB, NFKBIA, NDUFA8, ITLN1, and VNN1 (Figure 2G). Next, to enhance the accuracy and reliability of our findings, the GSE59867 dataset served as a platform to validate the expression levels of these seven genes. Notably, the expression levels of IL1RN, SERPINA1, CEBPB, NFKBIA, and VNN1 in patients with AMI were significantly elevated compared to those in the control cohort. Conversely, no substantial differences were observed between the two groups regarding the expression of NDUFA8 and ITLN1 (Figure 2H). Further corroboration from our RT-qPCR analysis, involving clinical samples from 7 AMI patients and 7 healthy controls, yielded results consistent with the aforementioned findings (Figure 2I). Consequently, the five identified genes—IL1RN, SERPINA1, CEBPB, NFKBIA, and VNN1—were utilized to develop a diagnostic model in the metadata cohort. Additionally, among the five candidate diagnostic signature genes, VNN1 is identified as a gene related to lipid metabolism, while IL1RN, SERPINA1, and CEBPB are associated with obesity. NFKBIA is a gene that relates to both obesity and lipid metabolism (Figure 2J).

### Diagnostic effectiveness of signature genes in AMI

**Figure 3:**
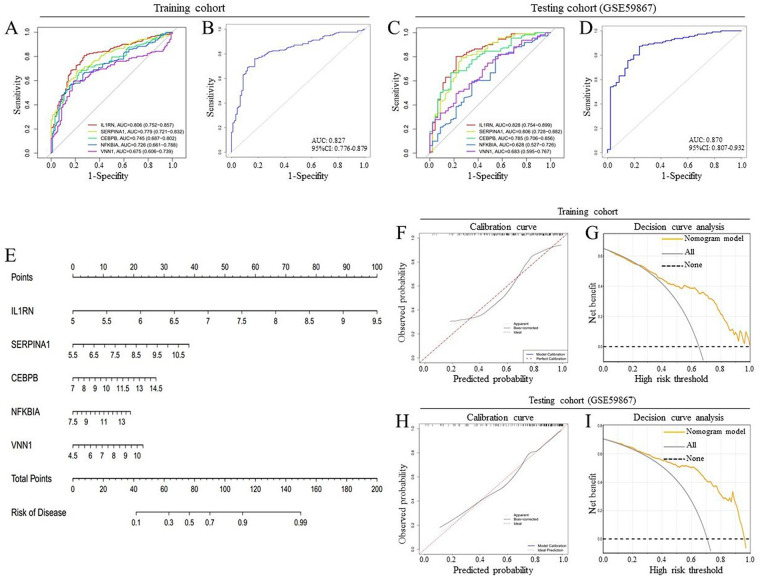
Assessment of signature genes and the diagnostic model for AMI diagnosis. **(A,B)** ROC curves for evaluating the diagnostic performance of signature genes in differentiating AMI patients from control subjects based on the training cohort. **(C,D)** ROC curves for evaluating the diagnostic performance of signature genes in distinguishing AMI patients from controls based on the testing cohort. **(E)** Nomogram model. **(F,G)** Calibration curve **(F)** and decision curve analysis (DCA) **(G)** to evaluate the predictive value and the clinical value in the training cohort. **(H,I)** Calibration curve **(H)** and DCA **(I)** to evaluate the predictive value and the clinical value in the testing cohort. ROC, receiver operating characteristic; AUC, area under the curve.

An analysis using ROC curve methodology was performed to assess the diagnostic performance of signature genes in differentiating AMI from the control group. As illustrated in Figure 3A, the findings revealed promising diagnostic values: an AUC of 0.806 (95% CI 0.752–0.857) for IL1RN, 0.779 (95% CI 0.721–0.832) for SERPINA1, 0.745 (95% CI 0.687–0.802) for CEBPB, 0.726 (95% CI 0.661–0.788) for NFKBIA, and 0.675 (95% CI 0.606–0.739) for VNN1. By integrating these five genes into a single variable, the AUC in the training cohort improved to 0.827 (95% CI 0.776–0.879) (Figure 3B). Additionally, a notable discriminatory capability was observed in the testing cohort (GSE59867), with the AUC values of 0.828 (95% CI 0.754–0.899) for IL1RN, 0.806 (95% CI 0.728–0.882) for SERPINA1, 0.785 (95% CI 0.706–0.856) for CEBPB, 0.628 (95% CI 0.527–0.726) for NFKBIA, and 0.683 (95% CI 0.595–0.767) for VNN1 (Figure 3C). Notably, the combination of the five signature genes produced an AUC of 0.870 (95% CI 0.807–0.932) (Figure 3D), which demonstrates that these biomarker features possess considerable diagnostic efficacy. Lastly, we evaluated the diagnostic performance of conventional biomarkers such as TNNT2, TNNI3, MB, CKB, and CKM in clinical settings for differentiating AMI from the control group, utilizing both the training and testing cohorts. Regardless of whether assessed individually or as a collective of five, these biomarkers exhibited significantly lower diagnostic capabilities compared to IL1RN, SERPINA1, CEBPB, NFKBIA, and VNN1 (Supplementary Figures S2A–D). These observations further reinforce the substantial clinical applicability potential of the biomarkers identified in our study. However, it is important to note that these analysis results are currently based solely on the mRNA expression levels of the target genes, and further research is required to conduct a comparative analysis of protein levels in the future.

### Signature genes interaction analyses

To delve deeper into the interaction and related functions of five specific signature genes, we utilized the GeneMANIA database to construct a protein-protein interaction (PPI) network for these genes, ultimately identifying 25 genes within the network (Supplementary Figure S3A). The signature genes were positioned in the inner circle, whereas the predicted genes were displayed in the outer circle. Their roles primarily revolved around responses to interleukin-1, leukocyte adhesion between cells, lipid storage, and lipid localization, aligning with prior research on the functional pathways associated with acute myocardial infarction. In the myocardium affected by infarction, the activation of the inflammatory cascade aids in the removal of dead cells, while also promoting matrix degradation and chamber dilation, which contributes to the onset of heart failure. Interleukin-1 plays a crucial role in the inflammatory response following infarction and fosters adverse dilative remodeling (41). The buildup of lipids leads to the accumulation of macrophages derived from monocytes within the intima, subsequently triggering a mild inflammatory response. Furthermore, the adhesion between leukocytes, platelets, and endothelial cells is essential in processes related to vascular inflammation and thrombus formation (42). In addition, Gene Set Enrichment Analysis (GSEA) revealed that the IL6-JAK-STAT3 signaling pathway was highly consistently enriched in the high CEBPB, NFKBIA, SERPINA1, VNN1, and IL1RN expression groups (Supplementary Figures S3B–F).

### Development of the diagnostic model based on signature genes

Signature genes were employed to develop a diagnostic model for gene-disease associations using a logistic regression algorithm, which resulted in the creation of a line chart, calibration map, and decision curve. The nomogram served to illustrate the model, incorporating the five DE-OLMRGs. The representation of each gene's fraction matched the proportions displayed in the nomogram. In the nomogram depicted (Figure 3E), IL1RN, SERPINA1, CEBPB, NFKBIA, and VNN1 acted as predictive factors for AMI. Elevated levels of five DE-OLMRGs were found to have a positive correlation with AMI diagnosis. Consequently, the total score derived from the five signature genes predicted the accuracy of the AMI diagnosis. The calibration plot for the nomogram regarding AMI diagnosis in the training cohort exhibited strong alignment between actual observations and nomogram predictions (Figure 3F), and the C-index for the nomogram model reached 0.8205 (95% CI = 0.7541–0.8850). The ROC curve analysis provided an AUC value of 0.827, demonstrating superior predictive accuracy relative to the aforementioned five DE-OLMRGs (Figure 3B). Meanwhile, the decision curve highlighted the nomogram model's potential clinical utility, suggesting that patients at high-risk thresholds, ranging from 0.3 to 1.0, could benefit significantly from this model (Figure 3G). Finally, for further validation of the prediction model's accuracy, we utilized the GSE59867 dataset. Notably, the AUC value (Figure 3D), calibration plot (Figure 3H), and decision curve (Figure 3I) exhibited positive characteristics, confirming that the diagnostic model comprised of five DE-OLMRGs is a reliable tool for diagnosing AMI.

### Identification of molecular subtype with differential immune infiltration features in AMI

Based on the median risk scores derived from the diagnostic model, all cases of AMI in both the training and testing cohorts (GSE59867) were divided into low-risk and high-risk categories. To evaluate the effectiveness of this risk stratification, analyses using PCA, t-SNE, and UMAP were performed on the diagnostic signature genes. Our findings indicated that the diagnostic model related to OLMRGs effectively distinguished patients into varying risk categories (Supplementary Figures S4A,B). We then proceeded to assess the expression levels of the signature genes across the control group, low-risk group, and high-risk group. As demonstrated in Supplementary Figure S5, all five signature genes exhibited significantly elevated expression levels in the high-risk group across both the training and testing cohorts. Furthermore, IL1RN and SERPINA1 displayed significant differences among the three groups within both the training and testing sets.

**Figure 4:**
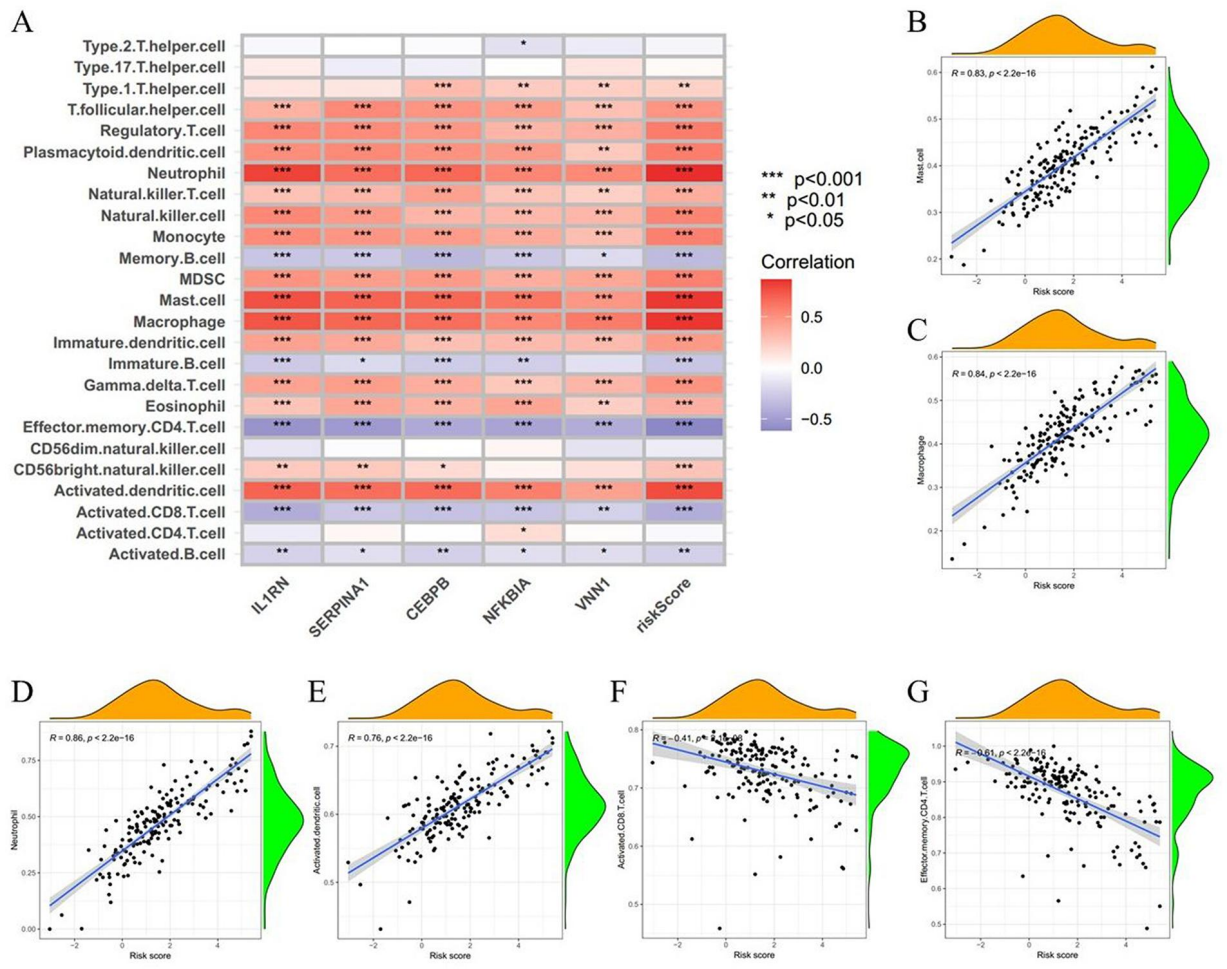
Analysis of the relationship between risk score and indicated immune cells. **(A)** Correlation between immune cells and five signature genes alongside the risk score. **(B–G)** The correlation analysis between the risk score and the proportion of mast cells **(B)**, macrophages **(C)**, neutrophils **(D)**, activated dendritic cells **(E)**, activated CD8T cells **(F)**, and effector memory CD4T cells **(G)** in AMI. *p < 0.05, **p < 0.01, ***p < 0.001.

Given that immune cell infiltration significantly influences ventricular remodeling and cardiac function following an infarction (43), we first analyzed the relationship between AMI risk score and immune cell infiltration. The results showed that the infiltration levels of various immune cells, such as mast cells, macrophages, neutrophils, activated dendritic cells, etc., were positively correlated with risk scores, while the infiltration levels of activated CD8T cells, effector memory CD4T cells, etc., were negatively correlated with risk scores (Figures 4A–G). In addition, an analysis involving 28 different types of immune cells was performed, with the score reflecting the extent of correlation. The results revealed that activated B cells and memory B cells exhibited the strongest synergistic interactions, whereas macrophages and central memory CD4T cells displayed significant competitive effects (Supplementary Figure S6).

**Figure 5:**
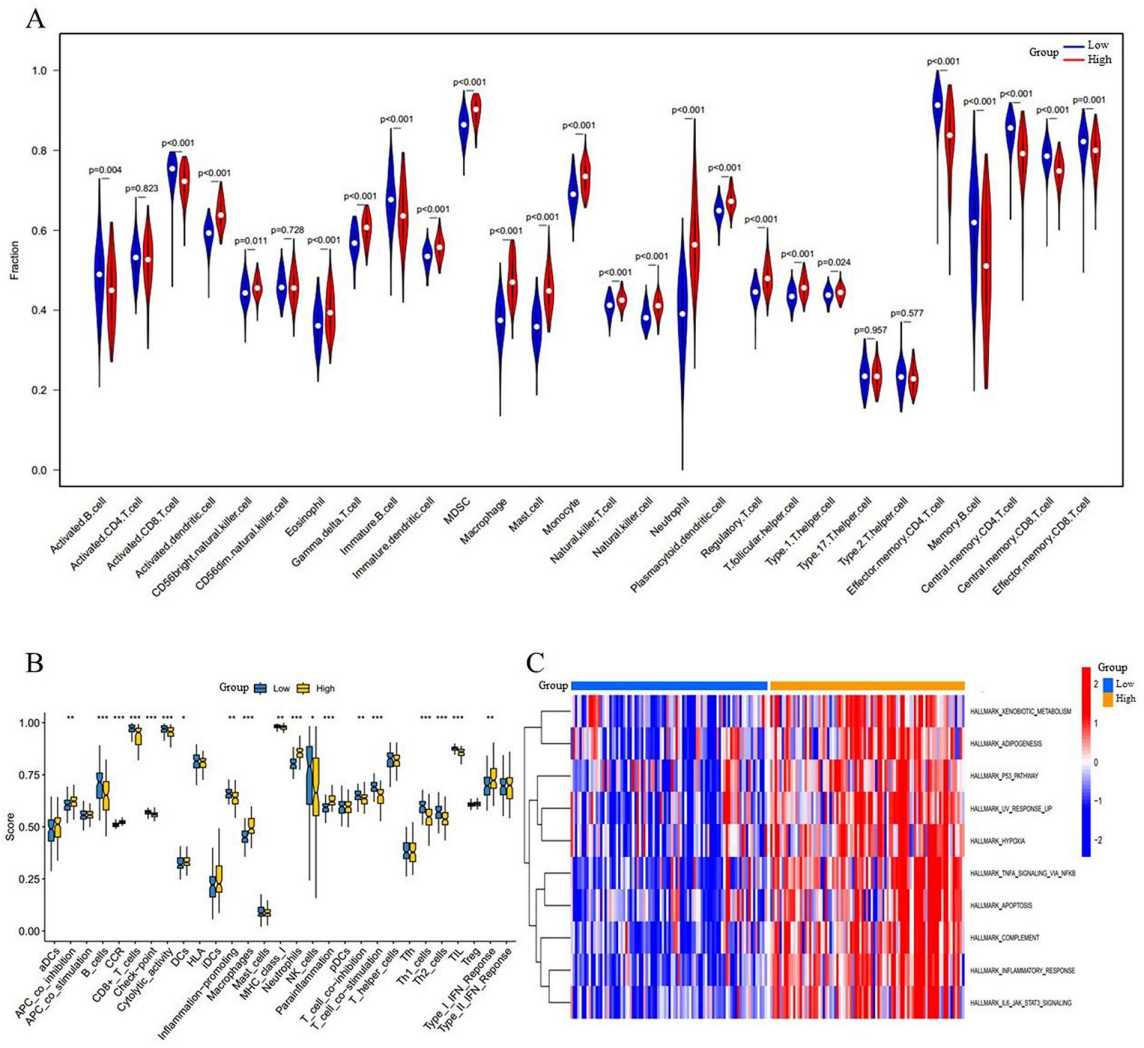
Comparison of the expression of immune cells and immune functions between low-risk and high-risk groups. **(A,B)** Differential analysis of immune cell infiltration **(A)** and 29 immune functions **(B)** across differential risk groups. **(C)** The results of gene set variation analysis (GSVA) for pathways based on the merged dataset including GSE62646, GSE66360, GSE60993, and GSE48060 databases. *p < 0.05, **p < 0.01, ***p < 0.001.

Then, we utilized the ssGSEA package to further investigate the differences in immune cell infiltration and immune functions between the high-risk and low-risk groups. Our analysis revealed that 24 out of 28 immune cell subsets displayed distinct infiltration patterns across these two risk categories. In comparison to the low-risk group, the high-risk group demonstrated a reduced proportion of several immune cells, including activated B cells, activated CD8T cells, immature B cells, effector memory CD4T cells, memory B cells, central memory CD4T cells, central memory CD8T cells, and effector memory CD8T cells. Additionally, the high-risk group exhibited an increased prevalence of activated dendritic cells, CD56 bright natural killer cells, eosinophils, gamma delta T cells, immature dendritic cells, MDSCs, macrophages, mast cells, monocytes, natural killer T cells, natural killer cells, neutrophils, plasmacytoid dendritic cells, regulatory T cells, T follicular helper cells, and type 1T helper cells (Figure 5A). Furthermore, we evaluated the relationship between immune cells and model signature genes across different risk groups, relying on data from the training cohort. In the high-risk group, CEBPB, NFKBIA, and SERPINA1 exhibited significant positive correlations with mast cells (*r* > 0.490, *p* < 0.001) and negative correlations with central memory CD4T cells (*r* < −0.360, *p* < 0.001). VNN1 showed a significant positive correlation with macrophages (*r* = 0.456, *p* = 1.46 × 10^−5^). IL1RN was significantly positively correlated with neutrophils (*r* = 0.666, *p* = 3.36 × 10^−12^) and negatively correlated with effector memory CD4T cells (*r* = −0.518, *p* = 3.73 × 10^−7^) (Supplementary Figures S7A–E). Conversely, in the low-risk group, CEBPB exhibited a significant positive correlation with natural killer T cells (*r* = 0.539, *p* = 8.43 × 10^−8^) and a negative correlation with memory B cells (*r* = −0.261, *p* = 0.015). VNN1 was significantly positively correlated with macrophages (*r* = 0.283, *p* = 8.33 × 10^−3^) and negatively correlated with effector memory CD4T cells (*r* = −0.301, *p* = 0.005). IL1RN was significantly positively correlated with neutrophils (*r* = 0.543, *p* = 1.09 × 10^−7^) and negatively correlated with central memory CD8T cells (*r* = −0.251, *p* = 0.020). NFKBIA showed a significant positive correlation with effector memory CD8T cells (*r* = 0.306, *p* = 0.004) and a negative correlation with central memory CD4T cells (*r* = −0.310, *p* = 0.004). SERPINA1 exhibited a significant positive correlation with T follicular helper cells (*r* = 0.534, *p* = 1.22 × 10^−7^) and a negative correlation with type 17T helper cells (*r* = −0.262, *p* = 0.015) (Supplementary Figures S8A–E). Moreover, a notable difference was observed among nineteen immune functions across the two risk groups, with the most significant being associated with APC co-stimulation, B cells, CCR, CD8+ T cells, checkpoints, cytolytic activity, dendritic cells (DCs), inflammation promotion, macrophages, MHC class I, neutrophils, NK cells, parainflammation, T cell co-inhibition, T cell co-stimulation, Th1 cells, Th2 cells, tumor-infiltrating lymphocytes (TIL), and type Ⅰ IFN response (Figure 5B). In addition, results from the GSVA enrichment analysis indicated that the high-risk group exhibited substantial enrichment in specific functions and pathways related to xenobiotic metabolism, adipogenesis, the P53 pathway, hypoxia, apoptosis, inflammatory response, and IL6-JAK-STAT3 signaling, among others (Figure 5C). This finding implies that these immune functions and pathways may play a crucial role during critical phases in the pathophysiology of AMI.

Finally, we paid particular attention to the relationship between various molecular subtypes of AMI and patient clinical outcomes. Utilizing the GSE59867 dataset, we found that the number of AMI patients in the high-risk group who subsequently developed heart failure (6 out of 8) was significantly higher than that in the low-risk group (3 out of 9). However, the chi-square test did not reveal any statistically significant differences (Supplementary Table S7). We hypothesize that this discrepancy may stem from errors introduced by the small sample size, indicating that further research is necessary to validate these findings.

## Discussion

Research indicates that the death rate due to cardiovascular diseases exceeds that of cancer by more than a factor of two, with more than half of these fatalities linked to AMI, positioning AMI as a significant threat to public health (44). Currently, detecting AMI commonly depends on changes in cardiac biomarkers. The standard biomarkers utilized in clinical practice include cardiac troponin T, cardiac troponin I, myoglobin, and creatine kinase-MB (CK-MB) (45). However, these biomarkers are mainly released from necrotic heart muscle cells within 2–4 h following the onset of AMI, and their levels may also increase in patients with chronic kidney disease, sepsis, heart failure, and thyroid issues, especially in older adults (46). Recently, advancements in both thrombolytic and interventional methods have greatly enhanced the prognosis for individuals suffering from AMI. Nevertheless, the early-stage diagnosis of AMI remains challenging; certain patients may present with atypical symptoms, which can result in treatment delays and a rise in AMI incidence (24). Consequently, it is crucial to discover effective diagnostic biomarkers and to develop diagnostic frameworks that can guide treatment and enhance patient outcomes.

The epidemic of global obesity is distinctly acknowledged, exhibiting a rise in obesity rates across most nations since the 1980s. Obesity is a direct contributor to emerging cardiovascular risk factors such as hypertension, type 2 diabetes, dyslipidemia, and sleep disorders. Furthermore, it fosters the onset of cardiovascular diseases and their mortality rates, independent of other risk factors. Recent findings emphasize abdominal obesity, identified through waist circumference measurements, as a distinct risk marker for cardiovascular disease that operates independently of body mass index (13). The processes of energy metabolism and inflammatory responses are crucial in the onset and progression of AMI, while lipid metabolism is essential for myocardial cell energy metabolism (47). However, prior studies did not investigate these two components simultaneously. To address this limitation, the present research uniquely connects obesity with lipid metabolism. Subsequently, a diagnostic gene signature derived from OLMRGs was developed, revealing encouraging outcomes for AMI diagnosis and risk assessment through a comprehensive analysis of transcriptional profiles alongside clinical data. This signature offers more precise predictions for AMI patients, with AUC values ranging from 0.776 to 0.932, and correlates with infiltration of immune cells.

Recent studies reveal that the infiltration of immune cells plays a vital role in the onset and progression of AMI (48, 49). These immune cells have specific functions related to different cell types that affect cardiac repair and unfavorable ventricular remodeling, influencing the prognosis of patients with AMI (50, 51). The crucial involvement of immune responses in the pathophysiology of AMI underscores the necessity for immune-related biomarkers in this condition. In the present research, we analyzed immune cell infiltration in AMI using the ssGSEA algorithm and examined the relationships between recognized signature genes and these infiltrating immune cells. Correlation analysis showed that IL1RN, SERPINA1, CEBPB, NFKBIA, and VNN1 exhibited positive correlations with T follicular helper cells, regulatory T cells, plasmacytoid dendritic cells, neutrophils, natural killer T cells, monocytes, myeloid-derived suppressor cells (MDSC), mast cells, macrophages, immature dendritic cells, gamma delta T cells, eosinophils, and activated dendritic cells, while they were negatively correlated with memory B cells, effector memory CD4T cells, activated CD8T cells, and activated B cells. Up to this point, a limited number of studies have explored this relationship. Furthermore, a significant increase in eosinophils, macrophages, mast cells, monocytes, natural killer T cells, MDSC, and neutrophils was observed in the high-risk group compared to the low-risk group. Currently, there is an increasing acknowledgment of the substantial role played by the innate immune system in the advancement of heart disease (52). Following AMI, various immune cells, such as neutrophils and monocytes, are mobilized to the heart, initiating a strong inflammatory response (53). The infiltration of neutrophils begins in the infarcted myocardium, where they can harm the extracellular matrix and provoke a wound healing response through the release of matrix-degrading enzymes (54). These infiltrating neutrophils negatively impact myocardial cells, resulting in reperfusion injury (55). A recent bioinformatics study revealed that neutrophils accumulate on the first day after AMI and may serve as a predictive marker for heart failure (56), which aligns with our findings. Macrophages, as a crucial component of the immune system, are essential for initiating, developing, and resolving inflammation following cardiac tissue damage (57). In addition to their established role in the immune response, macrophages engage in crosstalk with various other cells (including cardiomyocytes, fibroblasts, immune cells, and vascular endothelial cells) to manage post-myocardial infarction processes within cardiac tissue (57, 58). The exosomes secreted by macrophages have recently garnered significant interest, leading to a more nuanced understanding of macrophage functions (59). However, the specific functional roles of macrophages within the microenvironment of the infarcted heart, especially concerning their interactions with surrounding cells, remain ambiguous.

Previous studies have demonstrated that the genes featured in our risk signature are associated with acute myocardial infarction (AMI), including IL1RN (60), SERPINA1 (61), CEBPB (16), and NFKBIA (62). Specifically, IL1RN functions as a natural antagonist of IL-1 by attaching to the IL-1R1 receptor, which inhibits IL-1 signaling (63). Inflammation plays a crucial role in the pathophysiology of AMI, highlighted by the involvement of inflammatory mediators in the destabilization of plaques and injury to the myocardium (64). The adjustment of inflammation via IL1RN may affect the degree of myocardial harm and the remodeling that follows, supporting evidence that managing inflammation can lead to better outcomes in acute coronary syndromes (65).

Our findings also revealed that IL1RN possesses considerable diagnostic potential for AMI, as indicated by the ROC curve analysis in both the training cohort (AUC = 0.806, 95% CI: 0.752–0.857) and the testing cohort (AUC = 0.828, 95% CI: 0.754–0.899), suggesting that IL1RN is a reliable biomarker for AMI diagnosis. The Serpin peptidase inhibitor clade A member 1 (SERPINA1) gene encodes alpha 1-antitrypsin (AAT), which is the predominant serine protease inhibitor found in human plasma and has anti-inflammatory and immune-regulatory properties. Research has indicated that AAT levels are elevated in breast, gastric, and colorectal cancers (66–68). SERPINA1 influences fibronectin 1 through Snail in colorectal and gastric cancer, fostering epithelial-mesenchymal transitions that promote cancer advancement and metastasis (67, 68). Recently, Curjuric et al. observed a link between a genetic deficiency in SERPINA1 and an increased risk of cardiovascular issues (69). This protein's role in AMI may be connected to its ability to reduce proteolytic damage to tissues during ischemia-reperfusion episodes, which are crucial contributors to myocardial injury (70). Consequently, the modulation of protease activity by SERPINA1 might affect the extent of infarction and the subsequent healing processes. CEBPB serves as a vital transcription factor modulating the expression of genes involved in immune and inflammatory responses, and it is also significant in lipogenesis, gluconeogenesis, liver regeneration, and hematopoiesis (71). Wu et al. observed a significant increase in CEBPB levels in the bloodstream of patients with AMI, along with heightened expression in the peripheral blood and heart tissue of AMI-afflicted mice (16). The activation of CEBPB has been linked to the recruitment of inflammatory cells and the production of cytokines, which are critical to inflammation and scar development following an infarction (72). The potential mechanisms by which CEBPB influences AMI involve its ability to regulate the expression of genes associated with inflammation, ultimately impacting the healing and remodeling of myocardial tissue. NFKBIA, also known as NF-kappa B inhibitor alpha, is a protein that significantly influences the regulation of the NF-kappa B signaling pathway, which is vital for managing the expression of genes associated with immune responses, inflammation, and cell survival (73). The activation of the NF-κB pathway in response to myocardial ischemia plays a role in the production of inflammatory cytokines, cell apoptosis, and negative remodeling (74). The existence of NFKBIA indicates a regulatory mechanism that may modulate NF-κB-driven inflammation, thereby affecting the development of myocardial damage and subsequent repair processes. VNN1, also known as vascular noninflammatory molecule-1, functions as an enzyme that plays a role in the responses to oxidative stress and the regulation of cellular redox. It is well-documented that oxidative stress significantly contributes to myocardial damage during ischemia-reperfusion (75). The heightened expression of VNN1 is thought to indicate various inflammatory conditions (76) and chronic illnesses (77). The involvement of VNN1 in the regulation of oxidative stress responses corresponds with the mechanisms that affect cell survival, inflammation, and tissue remodeling following acute myocardial infarction (AMI). Despite all this, no existing studies have yet examined the combined diagnostic and molecular typing potential of these five signature genes for AMI.

In this investigation, we noted that the roles of the five marker genes primarily center on responses to interleukin-1, adhesion of intercellular leukocytes, as well as lipid storage and localization. The IL6-JAK-STAT3 signaling pathway exhibited significant enrichment in groups with high expression levels of CEBPB, NFKBIA, SERPINA1, VNN1, and IL1RN. This pathway, considered a crucial regulator of inflammation and cell survival, has a complex role in both the onset and progression of cardiovascular diseases (78, 79). Under normal physiological conditions, the pathway triggers the phosphorylation of STAT3 within myocardial cells, boosts the production of the anti-apoptotic protein Bcl-2, enhances myocardial cell resilience to injury, and aids in the repair of myocardial tissue while maintaining vascular endothelial stability (80). Conversely, in pathological states, persistent activation of these pathways serves as a major catalyst for disease advancement. In cases of atherosclerosis, IL-6 interacts with its receptor, activating JAK1/2 and allowing phosphorylated STAT3 to enter the nucleus where it influences the transcription of proinflammatory molecules like TNF-α and IL-1β, leading to increased inflammatory infiltration and lipid accumulation in the blood vessel walls (81, 82). During the acute phase of myocardial infarction, excessive pathway activation can result in the over-proliferation and fibrosis of myocardial cells, culminating in ventricular remodeling (83). Additionally, in the context of heart failure, metabolic disturbances mediated by STAT3 in myocardial cells and endothelial dysfunction exacerbate cardiac performance (84, 85).

The uniqueness of this research stems from our comprehensive examination of the relationship between OLMRGs and AMI, highlighting a significant approach to enrich existing knowledge by integrating OLMRGs into AMI risk evaluation. Understanding these mechanisms is vital for grasping the diagnosis and molecular classification of AMI, which contributes to a more profound insight into the progression of the condition. However, it is necessary to recognize that this research has specific limitations. Primarily, our examination relies significantly on publicly available datasets (GEO), which might not fully represent the diverse range of AMI patients on a global scale. An expansion of our research to encompass a broader and more diverse population, incorporating data from multiple centers and various ethnic backgrounds, would greatly enhance the relevance of our findings. Moreover, although we utilized the ComBat algorithm to mitigate batch effects across the datasets, these adjustments may not account for all sources of technical variation, potentially leading to minor biases in the gene expression data. Subsequent studies should concentrate on refining harmonization methods and incorporating additional datasets to validate our model's accuracy. In addition, a possible constraint of this research is the limited sample size in the RT-qPCR validation group (7 AMI patients compared to 7 controls), which could limit the statistical power and applicability of the validation outcomes. This modest sample size must be taken into account when analyzing the current results, and additional validation in larger and more varied independent groups is necessary. Finally, it is crucial to emphasize that the proposed diagnostic model related to OLMRGs is based on bioinformatics analysis, suggesting that considerable effort is still needed to bridge the gap between our findings and their practical implementation in clinical settings.

## Conclusion

A new obesity and lipid metabolism-related genes signature (OLMRGS) was developed for the early diagnosis and molecular typing of AMI, utilizing integrated bioinformatics analysis. We confirmed the efficacy of the diagnostic model using an independent dataset obtained from GEO. The OLMRGS demonstrated strong diagnostic capabilities for AMI and could potentially serve as a valuable biomarker for its diagnosis.

## Data Availability

The original contributions presented in the study are included in the article/Supplementary Material, further inquiries can be directed to the corresponding authors.

## References

[1] Murray CJL Barber RM Foreman KJ Ozgoren AA Abd-Allah F Abera SF Global, regional, and national disability-adjusted life years (DALYs) for 306 diseases and injuries and healthy life expectancy (HALE) for 188 countries, 1990–2013: quantifying the epidemiological transition. Lancet. (2015) 386:2145–91. 10.1016/S0140-6736(15)61340-X 26321261 PMC4673910

[2] Harjola V-P Lassus J Sionis A Køber L Tarvasmäki T Spinar J Clinical picture and risk prediction of short-term mortality in cardiogenic shock. Eur J Heart Fail. (2015) 17:501–9. 10.1002/ejhf.260 25820680

[3] Kapur NK Thayer KL Zweck E. Cardiogenic shock in the setting of acute myocardial infarction. Methodist Debakey Cardiovasc J. (2020) 16:16–21. 10.14797/mdcj-16-1-16 32280413 PMC7137623

[4] White HD Chew DP. Acute myocardial infarction. Lancet. (2008) 372:570–84. 10.1016/S0140-6736(08)61237-4 18707987

[5] Thygesen K Alpert JS White HD Jaffe AS Apple FS Galvani M Universal definition of myocardial infarction. Eur Heart J. (2007) 28:2525–38. 10.1093/eurheartj/ehm355 17951287

[6] Jaffe AS Babuin L Apple FS. Biomarkers in acute cardiac disease: the present and the future. J Am Coll Cardiol. (2006) 48:1–11. 10.1016/j.jacc.2006.02.056 16814641

[7] de Winter RJ Koster RW Sturk A Sanders GT. Value of myoglobin, troponin T, and CK-MBmass in ruling out an acute myocardial infarction in the emergency room. Circulation. (1995) 92:3401–7. 10.1161/01.CIR.92.12.3401 8521560

[8] de Lemos JA Drazner MH Omland T Ayers CR Khera A Rohatgi A Association of troponin T detected with a highly sensitive assay and cardiac structure and mortality risk in the general population. J Am Med Assoc. (2010) 304:2503–12. 10.1001/jama.2010.1768 PMC5621378 21139111

[9] Ge Y Wang TJ. Identifying novel biomarkers for cardiovascular disease risk prediction. J Intern Med. (2012) 272:430–9. 10.1111/j.1365-2796.2012.02589.x 22950687

[10] Braunwald E. Unstable angina and non-ST elevation myocardial infarction. Am J Respir Crit Care Med. (2012) 185:924–32. 10.1164/rccm.201109-1745CI 22205565

[11] Khan SS Ning H Wilkins JT Allen N Carnethon M Berry JD Association of body mass index with lifetime risk of cardiovascular disease and compression of morbidity. JAMA Cardiol. (2018) 3:280–7. 10.1001/jamacardio.2018.0022 29490333 PMC5875319

[12] Welsh A Hammad M Piña IL Kulinski J. Obesity and cardiovascular health. Eur J Prev Cardiol. (2024) 31:1026–35. 10.1093/eurjpc/zwae025 38243826 PMC11144464

[13] Powell-Wiley TM Poirier P Burke LE Després J-P Gordon-Larsen P Lavie CJ Obesity and cardiovascular disease: a scientific statement from the American Heart Association. Circulation. (2021) 143:e984–1010. 10.1161/CIR.0000000000000973 33882682 PMC8493650

[14] Stępień M Wlazeł RN Paradowski M Banach M Rysz M Misztal M Serum concentrations of adiponectin, leptin, resistin, ghrelin and insulin and their association with obesity indices in obese normo- and hypertensive patients—pilot study. Arch Med Sci. (2012) 8:431–6. 10.5114/aoms.2012.29518 22851996 PMC3400908

[15] Stępień M Stępień A Wlazeł RN Paradowski M Banach M Rysz J. Obesity indices and inflammatory markers in obese non-diabetic normo- and hypertensive patients: a comparative pilot study. Lipids Health Dis. (2014) 13:29. 10.1186/1476-511X-13-29 24507240 PMC3921991

[16] Wu J Luo J Cai H Zhu H Lei Z Lu Y Expression characteristics of lipid metabolism-related genes and correlative immune infiltration landscape in acute myocardial infarction. Sci Rep. (2024) 14:14095. 10.1038/s41598-024-65022-3 38890389 PMC11189450

[17] Fang Y Wu Y Liu L Wang H. The four key genes participated in and maintained atrial fibrillation process via reprogramming lipid metabolism in AF patients. Front Genet. (2022) 13:821754. 10.3389/fgene.2022.821754 35669184 PMC9163572

[18] Bangalore S Fayyad R Laskey R DeMicco DA Messerli FH Waters DD. Body-weight fluctuations and outcomes in coronary disease. N Engl J Med. (2017) 376:1332–40. 10.1056/NEJMoa1606148 28379800

[19] Jelenik T Flögel U Álvarez-Hernández E Scheiber D Zweck E Ding Z Insulin resistance and vulnerability to cardiac ischemia. Diabetes. (2018) 67:2695–702. 10.2337/db18-0449 30257974 PMC6245221

[20] Zhou Z Zhang S Ding S Abudupataer M Zhang Z Zhu X Excessive neutrophil extracellular trap formation aggravates acute myocardial infarction injury in apolipoprotein E deficiency mice via the ROS-dependent pathway. Oxid Med Cell Longev. (2019) 2019:1209307. 10.1155/2019/1209307 31249639 PMC6556343

[21] Xu J Yang Y. Potential genes and pathways along with immune cells infiltration in the progression of atherosclerosis identified via microarray gene expression dataset re-analysis. Vascular. (2020) 28:643–54. 10.1177/1708538120922700 32379583

[22] Gora M Kiliszek M Burzynska B. Will global transcriptome analysis allow the detection of novel prognostic markers in coronary artery disease and heart failure? Curr Genomics. (2013) 14:388–96. 10.2174/1389202911314090006 24396272 PMC3861890

[23] Wu Y Chen H Li L Zhang L Dai K Wen T Construction of novel gene signature-based predictive model for the diagnosis of acute myocardial infarction by combining random forest with artificial neural network. Front Cardiovasc Med. (2022) 9:876543. 10.3389/fcvm.2022.876543 35694667 PMC9174464

[24] Zhang N Zhou B Tu S. Identification of an 11 immune-related gene signature as the novel biomarker for acute myocardial infarction diagnosis. Genes Immun. (2022) 23:209–17. 10.1038/s41435-022-00183-7 36182975

[25] Wang Y Zhang X Duan M Zhang C Wang K Feng L Identification of potential biomarkers associated with acute myocardial infarction by weighted gene coexpression network analysis. Oxid Med Cell Longev. (2021) 2021:5553811. 10.1155/2021/5553811 34490057 PMC8418549

[26] Wu Z Wang D Tang C. Urinary sodium-potassium ratio as a genetic predictor of myocardial infarction. Coron Artery Dis. (2025) 36:561–8. 10.1097/MCA.0000000000001532 40326438

[27] Wang Y Xu X Shui X Ren R Liu Y. Molecular subtype identification of cerebral ischemic stroke based on ferroptosis-related genes. Sci Rep. (2024) 14:9350. 10.1038/s41598-024-53327-2 38653998 PMC11039763

[28] Hao Y Li R Fan C Gao Y Hou X wen W Identification and validation of mitophagy-related genes in acute myocardial infarction and ischemic cardiomyopathy and study of immune mechanisms across different risk groups. Front Immunol. (2025) 16:1486961. 10.3389/fimmu.2025.1486961 40114920 PMC11922711

[29] Leek JT Johnson WE Parker HS Jaffe AE Storey JD. The sva package for removing batch effects and other unwanted variation in high-throughput experiments. Bioinformatics. (2012) 28:882–3. 10.1093/bioinformatics/bts034 22257669 PMC3307112

[30] Shen L Huang H Li J Chen W Yao Y Hu J Exploration of prognosis and immunometabolism landscapes in ER+ breast cancer based on a novel lipid metabolism-related signature. Front Immunol. (2023) 14:1199465. 10.3389/fimmu.2023.1199465 37469520 PMC10352658

[31] Xu K Xia P Liu P Zhang X. A six lipid metabolism related gene signature for predicting the prognosis of hepatocellular carcinoma. Sci Rep. (2022) 12:20781. 10.1038/s41598-022-25356-2 36456877 PMC9715694

[32] She H Tan L Wang Y Du Y Zhou Y Zhang J Integrative single-cell RNA sequencing and metabolomics decipher the imbalanced lipid-metabolism in maladaptive immune responses during sepsis. Front Immunol. (2023) 14:1181697. 10.3389/fimmu.2023.1181697 37180171 PMC10172510

[33] Yu G Wang L-G Han Y He Q-Y. Clusterprofiler: an R package for comparing biological themes among gene clusters. Omics. (2012) 16:284–7. 10.1089/omi.2011.0118 22455463 PMC3339379

[34] Gustavsson EK Zhang D Reynolds RH Garcia-Ruiz S Ryten M. Ggtranscript: an R package for the visualization and interpretation of transcript isoforms using ggplot2. Bioinformatics. (2022) 38:3844–6. 10.1093/bioinformatics/btac409 35751589 PMC9344834

[35] Sanz H Valim C Vegas E Oller JM Reverter F. SVM-RFE: selection and visualization of the most relevant features through non-linear kernels. BMC Bioinformatics. (2018) 19:432. 10.1186/s12859-018-2451-4 30453885 PMC6245920

[36] Zhu X Yin T Zhang T Zhu Q Lu X Wang L Identification of immune-related genes in patients with acute myocardial infarction using machine learning methods. J Inflamm Res. (2022) 15:3305–21. 10.2147/JIR.S360498 35692951 PMC9174022

[37] Zhang P Feng J Rui M Xie J Zhang L Zhang Z. Integrating machine learning and single-cell analysis to uncover lung adenocarcinoma progression and prognostic biomarkers. J Cell Mol Med. (2024) 28:e18516. 10.1111/jcmm.18516 38958577 PMC11221317

[38] Suárez-Fariñas M Lowes MA Zaba LC Krueger JG. Evaluation of the psoriasis transcriptome across different studies by gene set enrichment analysis (GSEA). PLoS One. (2010) 5:e10247. 10.1371/journal.pone.0010247 20422035 PMC2857878

[39] Zheng W Zhou C Xue Z Qiao L Wang J Lu F. Integrative analysis of a novel signature incorporating metabolism and stemness-related genes for risk stratification and assessing clinical outcomes and therapeutic responses in lung adenocarcinoma. BMC Cancer. (2025) 25:591. 10.1186/s12885-025-13984-6 40170009 PMC11963273

[40] Lu F Zhou J Chen Q Zhu J Zheng X Fang N PSMA5 contributes to progression of lung adenocarcinoma in association with the JAK/STAT pathway. Carcinogenesis. (2022) 43:624–34. 10.1093/carcin/bgac046 35605971

[41] Saxena A Chen W Su Y Rai V Uche OU Li N IL-1 induces proinflammatory leukocyte infiltration and regulates fibroblast phenotype in the infarcted myocardium. J Immunol. (2013) 191:4838–48. 10.4049/jimmunol.1300725 24078695 PMC3822582

[42] Schwietzer MF Ebnet K. JAM-A: adhesion receptor and signaling regulator in atherosclerosis. Curr Atheroscler Rep. (2025) 27:75. 10.1007/s11883-025-01322-x 40728654 PMC12307491

[43] Nahrendorf M Swirski FK. Innate immune cells in ischaemic heart disease: does myocardial infarction beget myocardial infarction? Eur Heart J. (2016) 37:868–72. 10.1093/eurheartj/ehv453 26351395 PMC4789592

[44] Xiao H Cui X Liu L Lv B Zhang R Zheng T Identification and validation of lipid metabolism-related key genes as novel biomarkers in acute myocardial infarction and pan-cancer analysis. Aging. (2024) 16:9127–46. 10.18632/aging.205860 38787365 PMC11164520

[45] Morrow DA Cannon CP Jesse RL Newby LK Ravkilde J Storrow AB National academy of clinical biochemistry laboratory medicine practice guidelines: clinical characteristics and utilization of biochemical markers in acute coronary syndromes. Circulation. (2007) 115:e356–75. 10.1161/CIRCULATIONAHA.107.182882 17384331

[46] Wang C Jing Q. Non-coding RNAs as biomarkers for acute myocardial infarction. Acta Pharmacol Sin. (2018) 39:1110–9. 10.1038/aps.2017.205 29698386 PMC6289336

[47] Shah RV Steffen LM Nayor M Reis JP Jacobs DR Allen NB Dietary metabolic signatures and cardiometabolic risk. Eur Heart J. (2023) 44:557–69. 10.1093/eurheartj/ehac446 36424694 PMC10169425

[48] Weil BR Neelamegham S. Selectins and immune cells in acute myocardial infarction and post-infarction ventricular remodeling: pathophysiology and novel treatments. Front Immunol. (2019) 10:300. 10.3389/fimmu.2019.00300 30873166 PMC6400985

[49] Xu J-Y Xiong Y-Y Lu X-T Yang Y-J. Regulation of type 2 immunity in myocardial infarction. Front Immunol. (2019) 10:62. 10.3389/fimmu.2019.00062 30761134 PMC6362944

[50] Frangogiannis NG. Regulation of the inflammatory response in cardiac repair. Circ Res. (2012) 110:159–73. 10.1161/CIRCRESAHA.111.243162 22223212 PMC3690135

[51] Marchant DJ Boyd JH Lin DC Granville DJ Garmaroudi FS McManus BM. Inflammation in myocardial diseases. Circ Res. (2012) 110:126–44. 10.1161/CIRCRESAHA.111.243170 22223210

[52] Swirski FK Nahrendorf M. Cardioimmunology: the immune system in cardiac homeostasis and disease. Nat Rev Immunol. (2018) 18:733–44. 10.1038/s41577-018-0065-8 30228378

[53] Prabhu SD Frangogiannis NG. The biological basis for cardiac repair after myocardial infarction: from inflammation to fibrosis. Circ Res. (2016) 119:91–112. 10.1161/CIRCRESAHA.116.303577 27340270 PMC4922528

[54] Ma Y Yabluchanskiy A Lindsey ML. Neutrophil roles in left ventricular remodeling following myocardial infarction. Fibrogenesis Tissue Repair. (2013) 6:11. 10.1186/1755-1536-6-11 23731794 PMC3681584

[55] Timmers L Pasterkamp G de Hoog VC Arslan F Appelman Y de Kleijn DPV. The innate immune response in reperfused myocardium. Cardiovasc Res. (2012) 94:276–83. 10.1093/cvr/cvs018 22266751

[56] Yu C Zhou W. Peripheral neutrophils and naive CD4T cells predict the development of heart failure following acute myocardial infarction: a bioinformatic study. Rev Port Cardiol. (2021) 40:839–47. 10.1016/j.repc.2020.12.011 34857156

[57] Jian Y Zhou X Shan W Chen C Ge W Cui J Crosstalk between macrophages and cardiac cells after myocardial infarction. Cell Commun Signal. (2023) 21:109. 10.1186/s12964-023-01105-4 37170235 PMC10173491

[58] Peet C Ivetic A Bromage DI Shah AM. Cardiac monocytes and macrophages after myocardial infarction. Cardiovasc Res. (2020) 116:1101–12. 10.1093/cvr/cvz336 31841135 PMC7177720

[59] Liu S Chen J Shi J Zhou W Wang L Fang W M1-like macrophage-derived exosomes suppress angiogenesis and exacerbate cardiac dysfunction in a myocardial infarction microenvironment. Basic Res Cardiol. (2020) 115:22. 10.1007/s00395-020-0781-7 32112145

[60] Shao G. Integrated RNA gene expression analysis identified potential immune-related biomarkers and RNA regulatory pathways of acute myocardial infarction. PLoS One. (2022) 17:e0264362. 10.1371/journal.pone.0264362 35231061 PMC8887732

[61] Niu X Zhang J Zhang L Hou Y Pu S Chu A Weighted gene co-expression network analysis identifies critical genes in the development of heart failure after acute myocardial infarction. Front Genet. (2019) 10:1214. 10.3389/fgene.2019.01214 31850068 PMC6889910

[62] Liu W Li Y Zhang Y Li S Chen Y Han B Identification of biomarkers and immune infiltration in acute myocardial infarction and heart failure by integrated analysis. Biosci Rep. (2023) 43:BSR20222552. 10.1042/BSR20222552 37334672 PMC10329185

[63] Dinarello CA. Interleukin-1, interleukin-1 receptors and interleukin-1 receptor antagonist. Int Rev Immunol. (1998) 16:457–99. 10.3109/08830189809043005 9646173

[64] Bulnes JF González L Velásquez L Orellana MP Venturelli PM Martínez G. Role of inflammation and evidence for the use of colchicine in patients with acute coronary syndrome. Front Cardiovasc Med. (2024) 11:1356023. 10.3389/fcvm.2024.1356023 38993522 PMC11236697

[65] Abbate A Toldo S Marchetti C Kron J Van Tassell BW Dinarello CA. Interleukin-1 and the inflammasome as therapeutic targets in cardiovascular disease. Circ Res. (2020) 126:1260–80. 10.1161/CIRCRESAHA.120.315937 32324502 PMC8760628

[66] Chan HJ Li H Liu Z Yuan Y-C Mortimer J Chen S. SERPINA1 is a direct estrogen receptor target gene and a predictor of survival in breast cancer patients. Oncotarget. (2015) 6:25815–27. 10.18632/oncotarget.4441 26158350 PMC4694868

[67] Kwon CH Park HJ Choi JH Lee JR Kim HK Jo H-j Snail and serpinA1 promote tumor progression and predict prognosis in colorectal cancer. Oncotarget. (2015) 6:20312–26. 10.18632/oncotarget.3964 26015410 PMC4653007

[68] Yang J Xiong X Wang X Guo B He K Huang C. Identification of peptide regions of SERPINA1 and ENOSF1 and their protein expression as potential serum biomarkers for gastric cancer. Tumour Biol. (2015) 36:5109–18. 10.1007/s13277-015-3163-2 25677901

[69] Curjuric I Imboden M Bettschart R Caviezel S Dratva J Pons M Alpha-1 antitrypsin deficiency: from the lung to the heart? Atherosclerosis. (2018) 270:166–72. 10.1016/j.atherosclerosis.2018.01.042 29432934

[70] Zhao W Zhang X Rong J. SUMOylation as a therapeutic target for myocardial infarction. Front Cardiovasc Med. (2021) 8:701583. 10.3389/fcvm.2021.701583 34395563 PMC8355363

[71] Roy SK Hu J Meng Q Xia Y Shapiro PS Reddy SPM MEKK1 plays a critical role in activating the transcription factor C/EBP-beta-dependent gene expression in response to IFN-gamma. Proc Natl Acad Sci U S A. (2002) 99:7945–50. 10.1073/pnas.122075799 12048245 PMC123000

[72] Hua C-C Liu X-M Liang L-R Wang L-F Zhong J-C. Targeting the microRNA-34a as a novel therapeutic strategy for cardiovascular diseases. Front Cardiovasc Med. (2021) 8:784044. 10.3389/fcvm.2021.784044 35155600 PMC8828972

[73] Hayden MS Ghosh S. NF-κB, the first quarter-century: remarkable progress and outstanding questions. Genes Dev. (2012) 26:203–34. 10.1101/gad.183434.111 22302935 PMC3278889

[74] Liu Y Huang D Li Z Zhou L Cen T Wei B A plasma proteomic approach in patients with heart failure after acute myocardial infarction: insights into the pathogenesis and progression of the disease. Front Cardiovasc Med. (2023) 10:1153625. 10.3389/fcvm.2023.1153625 37265567 PMC10229768

[75] Lodrini AM Goumans MJ. Cardiomyocytes cellular phenotypes after myocardial infarction. Front Cardiovasc Med. (2021) 8:750510. 10.3389/fcvm.2021.750510 34820429 PMC8606669

[76] Sato W Horie Y Kataoka E Ohshima S Dohmen T Iizuka M Hepatic gene expression in hepatocyte-specific pten deficient mice showing steatohepatitis without ethanol challenge. Hepatol Res. (2006) 34:256–65. 10.1016/j.hepres.2006.01.003 16490391

[77] Yacobovich J Revel-Vilk S Tamary H. Childhood immune thrombocytopenia–who will spontaneously recover? Semin Hematol. (2013) 50(Suppl 1):S71–4. 10.1053/j.seminhematol.2013.03.013 23664522

[78] Wagner MA Siddiqui MA. The JAK-STAT pathway in hypertrophic stress signaling and genomic stress response. JAKSTAT. (2012) 1:131–41. 10.4161/jkst.20702 24058762 PMC3670293

[79] Fontes JA Rose NR Čiháková D. The varying faces of IL-6: from cardiac protection to cardiac failure. Cytokine. (2015) 74:62–8. 10.1016/j.cyto.2014.12.024 25649043 PMC4677779

[80] Cau R Saba L. Interlinking pathways: a narrative review on the role of IL-6 in cancer and atherosclerosis. Cardiovasc Diagn Ther. (2024) 14:1186–201. 10.21037/cdt-24-344 39790197 PMC11707487

[81] Papastamos C Antonopoulos AS Simantiris S Koumallos N Sagris M Theofilis P Interleukin-6 signaling in atherosclerosis: from molecular mechanisms to clinical outcomes. Curr Top Med Chem. (2023) 23:2172–83. 10.2174/1568026623666230718141235 37464827

[82] Pang Q You L Meng X Li Y Deng T Li D Regulation of the JAK/STAT signaling pathway: the promising targets for cardiovascular disease. Biochem Pharmacol. (2023) 213:115587. 10.1016/j.bcp.2023.115587 37187275

[83] Zhang S Liu X Goldstein S Li Y Ge J He B Role of the JAK/STAT signaling pathway in the pathogenesis of acute myocardial infarction in rats and its effect on NF-κB expression. Mol Med Rep. (2013) 7:93–8. 10.3892/mmr.2012.1159 23128561

[84] Roger I Milara J Montero P Cortijo J. The role of JAK/STAT molecular pathway in vascular remodeling associated with pulmonary hypertension. Int J Mol Sci. (2021) 22:4980. 10.3390/ijms22094980 34067108 PMC8124199

[85] Yang Q Ji H Modarresi Chahardehi A. JAK/STAT pathway in myocardial infarction: crossroads of immune signaling and cardiac remodeling. Mol Immunol. (2025) 186:206–17. 10.1016/j.molimm.2025.08.018 40882407

