# Supplementary material for: Machine learning-derived identification of an obesity and lipid metabolism-related genes signature for the diagnosis and molecular typing of acute myocardial infarction
Source: Front Cardiovasc Med. 2026 Mar 27;13:1694872. doi: 10.3389/fcvm.2026.1694872 (PMC13065660; doi:10.3389/fcvm.2026.1694872)
Supplement: Supplementary file 1 [file Table1.pdf]

Supplementary Table

Table S1 Details of the Gene Expression Omnibus (GEO) data sets

GEO ID	Control sample	AMI sample	Platform information
GSE48060	21	31	GPL570
GSE60993	7	7	GPL6884
GSE66360	50	49	GPL570
GSE62646	14	28	GPL6244
GSE59867	46	111	GPL6244
