# Supplementary material for: Machine learning-derived identification of an obesity and lipid metabolism-related genes signature for the diagnosis and molecular typing of acute myocardial infarction
Source: Front Cardiovasc Med. 2026 Mar 27;13:1694872. doi: 10.3389/fcvm.2026.1694872 (PMC13065660; doi:10.3389/fcvm.2026.1694872)
Supplement: Supplementary file 4 [file Table4.pdf]

Supplementary Table

Table S4. Summary of clinical data

Clinical data	AMI (n=7)	Control (n=7)	P value
Age (year) [a]	61.8 ± 5.28	60.39 ± 2.64	0.538
Gender [b]			
Male (n %)	3 (42.9%)	4 (57.1%)	1.000
Female (n %)	4 (57.1%)	3 (42.9%)	
BMI (kg/m^2) [a]	25.03 ± 1.06	24.61 ± 1.10	0.482
Hyperlipemia (n %) [b]	4 (57.1%)	2 (28.6%)	0.592
Hypertension (n %) [b]	3 (42.9%)	2 (28.6%)	1.000
Smoking (n %) [b]	4 (57.1%)	3 (42.9%)	1.000
History of diabetes (n %) [b]	2 (28.6%)	2 (28.6%)	1.000
Family genetic history (n %) [b]	3 (42.9%)	1 (14.3%)	0.559

Notes: a, p values were calculated by t-test; b, p values were calculated by the chi-square test (Fisher's Exact Test)
