# Supplementary material for: Machine learning-derived identification of an obesity and lipid metabolism-related genes signature for the diagnosis and molecular typing of acute myocardial infarction
Source: Front Cardiovasc Med. 2026 Mar 27;13:1694872. doi: 10.3389/fcvm.2026.1694872 (PMC13065660; doi:10.3389/fcvm.2026.1694872)
Supplement: Supplementary file 5 [file Table5.pdf]

Supplementary Table

Table S5 Oligo sequences used in quantitative real-time PCR

Species	Gene	Primer Sequence	
Homo sapiens	IL1RN	Forward	GCAAGCCTTCAGAATCTGGG
		Reverse	GGACAGGCACATCTTCCCTC
Homo sapiens	SERPINA1	Forward	ACCAGTCCAACAGCACCAAT
		Reverse	TGGTTGAGGGTACGGAGGAG
Homo sapiens	NFKBIA	Forward	AGCTCCGAGACTTTCGAGGA
		Reverse	CACCAAAAGCTCCACGATGC
Homo sapiens	NDUFA8	Forward	TGGTCAACAAGTGTGCTTTGG
		Reverse	TCACTTTGGTGACCTTTGACAG
Homo sapiens	VNN1	Forward	GTACCCAAGGAGCCTGAGATT
		Reverse	ATGCCCATAGCCCAAGCTGA
Homo sapiens	CEBPB	Forward	GCACAGCGACGAGTACAAGA
		Reverse	TGCTTGAACAAGTTCCGCAG
Homo sapiens	ITLN1	Forward	AGCACTGGAGAAACAGCTCC
		Reverse	GGGCGTCGCCAAAATCATAG
Homo sapiens	GAPDH	Forward	TCCAAAATCAAGTGGGGCGA
		Reverse	TGATGACCCTTTTGGCTCCC
