# Supplementary material for: Machine learning-derived identification of an obesity and lipid metabolism-related genes signature for the diagnosis and molecular typing of acute myocardial infarction
Source: Front Cardiovasc Med. 2026 Mar 27;13:1694872. doi: 10.3389/fcvm.2026.1694872 (PMC13065660; doi:10.3389/fcvm.2026.1694872)
Supplement: Supplementary file 7 [file Table7.pdf]

Supplementary Table

Table S7 Association between subtype and heart failure (HF)

Subtype	No-HF (n=8)	HF (n=9)	Asymptotic Significance (2-sided)
high-risk	2	6	0.218
low-risk	6	3	
